# Research on graph theory segmentation method and dataset construction for connected handwritten digits

**DOI:** 10.1371/journal.pone.0360920

**Published:** 2026-10-29

**Authors:** Zhilai Zhang, Yang Zhang, Yang Wang

**Affiliations:** 1 Hebei Key Laboratory of Optical Fiber Biosensing and Communication Devices, Institute of Information Technology, Handan University, Handan, China; 2 School of Information Science and Engineering, Hebei University of Science and Technology, Shijiazhuang, Hebei, China; University of Baghdad, IRAQ

## Abstract

Handwritten digit recognition has considerable potential for automating document analysis. However, in real-world scenarios, character adhesion is a critical constraint on the performance of traditional automated educational scoring systems. To address the challenge of segmenting touching handwritten digits, this research presents a systematic investigation from both data and methodological perspectives. First, a standardized, category-balanced Two-digit Touching image dataset is constructed. By covering all 100 combinations from “00” to “99,” it simulates realistic adhesion patterns by defining a maximum overlap ratio and introducing random misalignment, thereby providing a fair benchmark for algorithm evaluation. On the methodological front, this paper proposes a “cutting domain” graph-theoretic segmentation framework. This framework decouples the adhesion segmentation problem into two stages: constructing a structured, low-cost search space (the cutting domain) with a threshold-free growth algorithm, and then transforming the segmentation task into an optimal path search on a graph. Within this cutting domain, the globally optimal Dijkstra’s algorithm and a family of direction-biased A* heuristic algorithms are implemented and compared with traditional methods (the Vertical Projection Method and the Watershed algorithm) as well as a deep supervised segmentation baseline (U-Net). Experimental results show that the proposed graph-theoretic segmentation method significantly outperforms traditional methods. Among the methods tested, the Left-Biased A* algorithm achieves the highest recognition accuracy of 93.13% on the test set, an improvement of over 5 percentage points over traditional methods. Ablation experiments confirm the critical role of connectivity constraints. Further evaluation on real-world handwritten digit strings (the CVL dataset) confirms the framework’s generalization, maintaining 91.4% accuracy, whereas a cross-paradigm comparison shows a supervised U-Net baseline collapsing from 95.90% to 53.05% under the same domain shift. This study provides a standardized evaluation benchmark and novel methodological insights for segmenting touching characters, thereby advancing document image analysis technology.

## 1 Introduction

### 1.1 Research background and significance

Handwritten digit recognition is a well-established task in document analysis and pattern recognition, with broad applications in practical scenarios such as postal mail sorting, bank check processing, automated educational scoring, and historical document digitization [1–3]. However, in real-world settings, handwritten digits often exhibit inter-character adhesion, overlap, or distortion due to individual writing habits, low-quality paper, or scanning noise. These issues severely degrade the performance of traditional optical character recognition systems, which fail to reliably segment touching digits from images [4–6]. Resolving the segmentation of touching handwritten digits is thus a pivotal prerequisite for advancing the automation of document analysis in unconstrained environments.

### 1.2 Challenges in touching digit segmentation

Existing methods for touching digit segmentation can be broadly categorized into three types, each with inherent limitations. The first category includes traditional heuristic-based methods, such as vertical projection [5,7,8], connected component analysis [9–11], and the watershed algorithm [12,13]. These methods are computationally efficient and effective for simple touching cases (e.g., slight side-by-side adhesion). Still, they are prone to over- or under-segmentation when confronted with complex adhesion patterns (e.g., irregular overlap or penetration). The second category comprises deep learning-based segmentation networks, represented by state-of-the-art models such as U-Net and Mask R-CNN [14–17]. While these methods excel at learning complex spatial features from data, they rely heavily on large-scale, high-quality annotated datasets and generalize poorly to unseen adhesion patterns (e.g., rare digit combinations or unusual writing styles). The third category consists of end-to-end recognition methods, such as sequence models based on connectionist temporal classification or attention mechanisms [18–20]. Such methods bypass explicit segmentation yet are prone to model redundancy and overfitting on short-digit string tasks.

Notably, graph-theoretic methods (e.g., shortest-path-based segmentation) have demonstrated inherent advantages for global optimization in image segmentation [21–23]. However, traditional graph-based methods (such as Graph Cut and Random Walk) typically couple graph construction and optimal path search within an energy minimization framework, whose energy functions involve hyperparameters that require manual tuning. This coupled design and parameter dependence lead to subjective tuning and limited generalization, making these methods less adaptable to the specific domain of handwritten digit adhesion segmentation, where adhesion patterns are highly variable.

Furthermore, existing public datasets (e.g., MNIST [24], KMNIST [25], NIST SD19 [26]) primarily serve as benchmarks for single-digit recognition and lack standardized datasets tailored to touching digit pairs. Most existing touching digit datasets either cover a limited range of digit combinations, lack quantitative descriptions of adhesion patterns (e.g., overlap ratio, misalignment degree), or suffer from category imbalance. This shortage hinders fair comparisons among segmentation algorithms and slows the field’s iterative progress. Consequently, there is an urgent need to construct a large-scale, standardized dataset of touching handwritten digit pairs that covers diverse adhesion patterns and provides explicit statistical metrics for quantitative evaluation.

### 1.3 Main contributions and structure of this paper

To address the aforementioned challenges (i.e., the lack of standardized datasets and the limitations of existing segmentation methods), this paper proposes a decoupled graph-theoretic framework for segmenting touching handwritten digits and constructs a corresponding benchmark dataset. The core contributions of this study are outlined as follows:

- **Construction of a standardized two-digit touching segmentation (TDTS) dataset:** Built on the MNIST dataset, TDTS encompasses all 100 digit combinations from “00” to “99” and simulates diverse adhesion patterns (e.g., side-by-side overlap, vertical misalignment). Key metrics, including adhesion degree (quantified by overlap ratio) and category balance, are systematically controlled and measured, providing a refined, reproducible benchmark for evaluating touch digit segmentation research.
- **Proposal of a “cutting domain”-based decoupled graph-theoretic segmentation framework:** This framework decomposes the segmentation task into two independent stages to avoid the limitations of coupled designs. First, a threshold-free region-growing algorithm constructs a structured search space (i.e., the “cutting domain”), ensuring low computational cost and adaptability to diverse adhesion patterns. Second, Dijkstra’s algorithm is used within this domain to find the globally optimal cutting path. At the same time, a family of direction-biased A* heuristic algorithms is designed to handle complex penetration-type adhesion scenarios. This decoupled design achieves both efficiency and segmentation precision.
- **Systematic validation of the proposed framework:** Comparative experiments and ablation studies are conducted to demonstrate its superiority. The proposed method achieves a segmentation-based recognition accuracy of approximately 93.1%, outperforming traditional baseline methods (with an average accuracy of 88.0%) by over 5 percentage points. Ablation experiments further clarify the influence mechanisms of key factors (e.g., spatial connectivity constraints, neighborhood range selection), providing empirical guidance for the design of graph-theoretic segmentation algorithms.
- **Validation on real-world data and comparison with a deep learning baseline:** The framework is further evaluated on real-world touching digit strings from the CVL dataset, maintaining 91.4% accuracy, and compared against a supervised U-Net baseline, which collapses to 53.05% under the same domain shift, demonstrating the generalization advantage of the proposed unsupervised graph-theoretic approach.

## 2 Related work

Segmenting touching handwritten digits is a pivotal challenge in document image analysis. Existing methods can be primarily categorized into two classes: those that require explicit cutting and those that circumvent explicit segmentation [27]. This chapter reviews representative works and their limitations within these two categories and analyzes the current state of relevant datasets. The goal is to clarify the research bottlenecks in this field and highlight the innovative aspects of the proposed work.

### 2.1 Segmentation methods

Existing methods for touching-digit segmentation can be broadly grouped into three categories: heuristic-based segmentation, deep learning-based segmentation, and end-to-end recognition.

Segmentation-oriented methods adopt a “segment-then-recognize” pipeline [28]. The vertical projection method cuts by identifying valleys in the pixel-density histogram, but it often fails when characters are tightly adhered or overlapping [29]. The traditional watershed algorithm is sensitive to hyperparameters and image noise, usually resulting in severe over-segmentation [30]. To enhance robustness, existing studies frequently integrate multi-strategy fusion (e.g., combining projection analysis with contour feature extraction) and dynamic programming to select the optimal cutting path [31–33]. However, the performance of these methods depends heavily on manually designed rules and thresholds, leading to limited generalization and high computational complexity in unconstrained scenarios [34].

Deep learning-based segmentation methods learn per-pixel class predictions through encoder–decoder architectures, achieving near-perfect accuracy within their training distribution [35]. U-Net and its variants, as well as deep autoencoder-based structures, have been applied to touching-character segmentation in handwritten digit strings and document images [9]. However, two structural limitations remain: first, training requires large-scale pixel-level annotation, which for touching handwritten characters can only be obtained through synthetic generation or manual labeling; second, generalization is bounded by the training distribution—once the target domain deviates in handwriting style, stroke width, or adhesion morphology, performance degrades sharply.

End-to-end recognition methods (e.g., CNN-RNN-CTC models and their attention-based variants) aim to bypass explicit segmentation and directly output character sequences from input images [36]. Although these methods have shown promising performance on variable-length digit strings, their black-box nature makes the implicit segmentation process uninterpretable. Moreover, they are prone to overfitting in small-sample settings, complicating the diagnosis and optimization of pixel-level segmentation errors.

In summary, when confronted with complex and variable adhesion patterns (e.g., irregular overlap or penetration), existing methods struggle to achieve a satisfactory balance among segmentation accuracy, generalization capability, and computational efficiency—this constitutes a key motivation for the proposed work.

### 2.2 Graph-theoretic segmentation methods

Graph theory provides a robust global optimization framework for image segmentation: it models images as graphs (with pixels as nodes and inter-pixel relationships as edges) [37]. It solves segmentation tasks via optimization objectives such as min-cut or shortest path. In the broader field of image segmentation, graph-theoretic methods have demonstrated inherent advantages in capturing global structural information. For instance, Graph Cut achieves segmentation by minimizing an energy function defined over pixels, but it requires interactive initialization and cumbersome tuning of energy function hyperparameters [38]. Random Walk performs pixel labeling via probabilistic diffusion with similarity weights, but it also suffers from sensitivity to weight parameter settings. Particularly noteworthy are shortest-path-based methods (e.g., Dijkstra’s algorithm and the A* heuristic), which can locate the optimal boundary between two target points under a global optimality criterion. These methods have achieved precise and efficient results in tasks such as medical image contour extraction and road network segmentation.

However, graph-theoretic methods have not been systematically applied to segmenting touching handwritten digits. The core challenge is constructing a graph model that accurately captures the adhesive structure of handwritten digits while remaining compatible with classical shortest-path search algorithms. Most existing studies use graph theory solely as a post-processing tool for segmentation refinement, lacking a comprehensive segmentation framework grounded in graph modeling. This leads to two critical limitations: first, graph structure construction (i.e., node/edge definition) and optimal cut search are typically coupled within a complex multi-parameter energy function, making hyperparameter tuning difficult and generalization to diverse adhesion patterns poor; second, mature and efficient optimal path algorithms (e.g., Dijkstra’s and A*) cannot be directly applied, as they require a predefined, structured, and discrete search space as input.

Therefore, exploring a decoupled framework—first constructing a threshold-free discrete “cutting domain” as the dedicated search space, then applying classical shortest-path algorithms to localize the optimal cut—holds significant innovative potential for addressing the aforementioned limitations.

### 2.3 Handwritten digit datasets: The gap from single characters to touching pairs

Current mainstream public datasets, such as MNIST [24], KMNIST [25] and NIST SD19 [26], are designed exclusively for single-character recognition tasks and thus cannot support the evaluation of touching-digit segmentation algorithms. Real-world datasets for digit-string recognition (e.g., CVL HDS, ORAND-CAR) provide a platform for system-level performance assessment [39–41], but the touching patterns in these datasets are naturally occurring. As a result, they exhibit inherent drawbacks, including unbalanced digit-combination distributions and uncontrollable adhesion degrees (e.g., overlap ratio, spatial misalignment), which make them inadequate for fine-grained, attributable performance evaluation and quantitative comparison of segmentation algorithms.

To fill this gap in the evaluation chain, there is an urgent need for a standardized, large-scale benchmark dataset of touching-digit pairs—characterized by controllable adhesion patterns, quantifiable adhesion metrics, a balanced distribution of digit combinations, and precise annotations. This requirement directly motivates the construction of the Two-digit Touching Segmentation dataset proposed in the Chapter Materials.

### 2.4 Chapter summary

This chapter has reviewed the state of the art in handwritten digit segmentation. Traditional methods are limited by poor adaptability to complex adhesion patterns; graph-theoretic methods, despite their global optimization advantages, remain underutilized due to the lack of a suitable modeling and solution framework; meanwhile, existing datasets fail to meet the demands of fine-grained, reproducible evaluation of segmentation algorithms. In response to these bottlenecks, this paper proposes a decoupled “cutting domain”-based graph-theoretic segmentation framework (integrating threshold-free search space construction and classical shortest path algorithms) and constructs the standardized TDTS dataset, aiming to advance research in this field.

## 3 Materials

This chapter focuses on constructing a standardized benchmark dataset for two-digit touching image segmentation. Designed to provide a testing platform with controllable adhesion patterns, minimized interfering factors, and fair evaluation conditions, this dataset enables accurate quantification and comparison of the core performance of various segmentation algorithms—including the proposed method. It thereby establishes an objective, reproducible benchmark for related research in this field.

### 3.1 Design objectives and core principles

To ensure that performance differences among algorithms directly reflect the merits of their segmentation logic, rather than being confounded by inherent dataset biases or irrelevant degradation factors, the construction of this dataset adheres to the following four core design principles:

- **Standardization of adhesion morphology:** A maximum overlap ratio of 30% is explicitly defined, and random spatial misalignment is introduced. This design simulates handwriting adhesion patterns while maintaining control over the generation process, laying a foundation for quantitative comparison of algorithms.
- **Purification of the evaluation environment:** The introduction of noise is strictly controlled, and complex real-world degradation factors (e.g., paper texture, uneven illumination) are stripped away. This ensures that the evaluation focuses exclusively on the algorithms’ ability to handle the adhesion structure itself.
- **Comprehensive coverage and class balancing:** All 100 digit combinations from “00” to “99” are fully covered. A targeted sampling strategy is employed to balance the number of samples per class, preventing evaluation bias caused by model over-adaptation to data imbalance.
- **process compatibility and fairness:** The storage format is kept consistent with MNIST. Specific pixel-level fusion rules and a dedicated label encoding scheme are applied during image generation, thereby eliminating discrepancies in the underlying pixel statistics and label space between single-character and touching-pair images.

### 3.2 Generation of a single two-digit touching image

Generating a single two-digit touching image is a parametric process. Its core operation involves synthesizing two preprocessed MNIST compact characters (i.e., 20×20-pixel core-stroke regions extracted from raw MNIST images) into a single image with standardized adhesion properties. As illustrated in Fig 1, this process mainly includes four steps: generating a noisy background canvas, fixing the left character at a predefined position, randomly placing the right character within a specified region, and performing pixel fusion according to dedicated rules.

**Fig 1:**
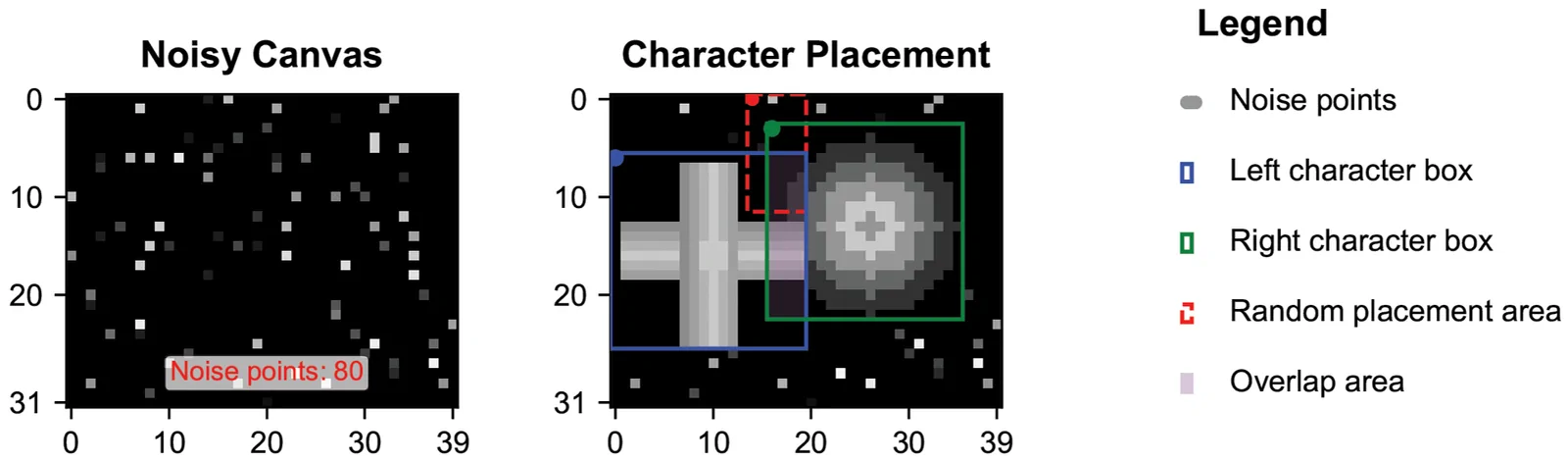
Generation workflow for a single touching digit pair image.

#### 3.2.1 Geometric model.

This study uses MNIST compact characters as the geometric basis for positional calculation, ensuring that the theoretical rectangular overlap ratio effectively reflects the actual pixel-level adhesion. To meet the design requirement of a 30% maximum overlap ratio and random spatial misalignment, the synthetic canvas is set to 40×32 pixels. The left character is fixed at coordinates (0, 6) (top-left corner). The top-left corner coordinates (x, y) of the right character are uniformly randomly generated within the region x∈[14,20] and y∈[0,12], hereafter referred to as the Right Character Placement Region. This approach guarantees both diversity and controllability of the generated adhesion morphologies.

#### 3.2.2 Noise and fusion model.

A “noise-first, then overlay” strategy is adopted for image generation. First, a background canvas with predefined noise is generated. Subsequently, character pixels are fused in a region-specific manner: for non-overlapping areas, character pixels directly overwrite the background; for overlapping areas, the pixel value is determined by taking the maximum grayscale value of the corresponding pixels from the left and right characters. This strategy preserves the integrity of character strokes in non-overlapping regions while simulating the visual appearance of genuine handwriting adhesion in overlapping zones, thus constructing a controlled yet realistic test environment for segmentation algorithms.

### 3.3 Dataset construction pipeline

The overall pipeline for dataset construction is illustrated in Fig 2 and consists of three core stages: data preparation, batch image generation, and dataset library storage.

**Fig 2:**
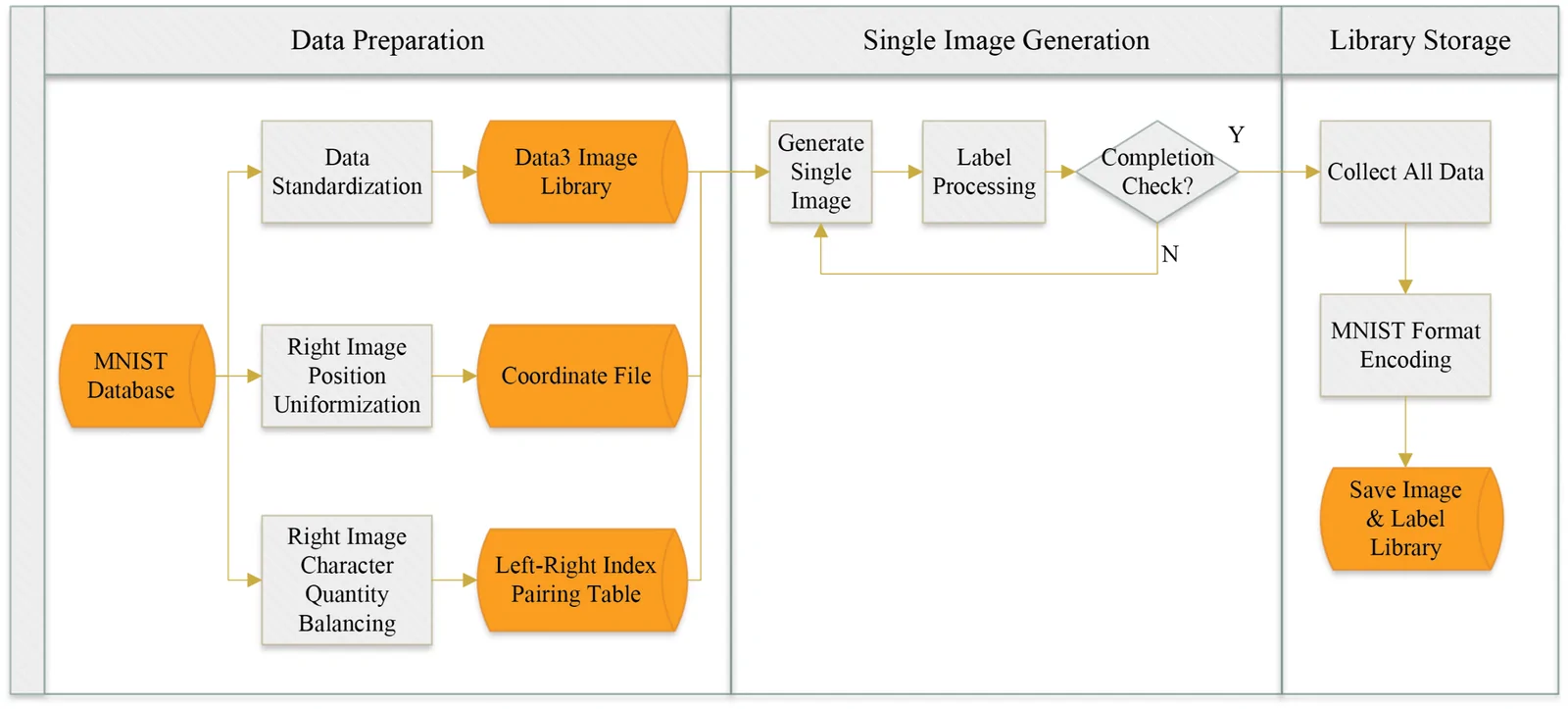
Workflow for constructing the touching digit dataset.

#### 3.3.1 Data source and preprocessing.

The “Data3” directory in the workflow stores preprocessed MNIST compact characters, which serve as the fundamental building blocks for constructing the two-digit touching image dataset. These compact characters are also used to generate input data for the subsequent single-character recognition model, ensuring consistency in data preprocessing across the entire experimental pipeline.

#### 3.3.2 Geometric control.

A dedicated coordinate file records the top-left corner coordinates (x, y) of the right character for each image pair. These coordinates precisely control the overlap ratio and spatial misalignment pattern between the two characters, enabling quantitative analysis of algorithm performance under different adhesion conditions.

#### 3.3.3 Sample balancing strategy.

The full MNIST dataset is directly used for the left characters. For the right characters, a balanced sampling strategy is implemented. For each digit class, the number of samples in the single-character training set and the single-character test set is adjusted, respectively, to the predefined mean values (6,000 for the single-character training set and 1,000 for the single-character test set). If the available samples for a class are insufficient, sampling with replacement is performed; if the number exceeds the target, random downsampling is applied. This results in a uniformly distributed index pool for the right characters, ensuring class balance across the dataset.

#### 3.3.4 Label encoding rule.

The label of a two-digit touching image is calculated using Formula (1). This encoding scheme avoids conflicts between the generated two-digit labels and the original MNIST single-digit labels (0–9), ensuring uniqueness and backward compatibility within the label space:


$$\begin{array}{c} \text{label}=L\times 10+R, \end{array}$$ (1)

where L and R represent the labels of the left and right characters, respectively.

### 3.4 Dataset characteristics and statistical analysis

The final constructed dataset consists of 60,000 training-source images (Train-derived Digit Touching Set: TDTS-TR) and 10,000 test-source images (Test-derived Digit Touching Set: TDTS-TS). Its fundamental attributes are fully compatible with MNIST, facilitating direct integration into existing deep learning training pipelines without additional format conversion.

#### 3.4.1 Class balance analysis.

To validate the effectiveness of the balancing strategy, Mean Absolute Deviation (MAD) and Coefficient of Variation (CV) are adopted as evaluation metrics, with comparisons made against the original non-balanced scheme (simulated via the Monte Carlo method). As shown in Table 1, the balanced strategy significantly improves class balance: TDTS-TR and TDTS-TS under the balanced strategy exhibit lower MAD and CV values compared to the simulated original strategy, confirming the effectiveness of the proposed strategy.

**Table 1:** Class balance metrics.

Dataset	Strategy	MAD	CV
TDTS-TR	Balanced strategy	28.64	0.0631
	Original strategy (Simulated)	39.33±1.14	0.0844±0.0013
TDTS-TS	Balanced strategy	8.64	0.1110
	Original strategy (simulated)	9.75±0.49	0.1230±0.0053

#### 3.4.2 Adhesion morphology statistics.

Quantitative statistical analysis of adhesion degree is performed on two-digit touching images, distinguishing between “Rectangular Adhesion” (the overlap ratio of bounding boxes calculated from generation coordinates) and “Actual Adhesion” (the true adhesion rate based on pixel-level overlap). The statistical results in Table 2 indicate that the distribution of rectangular adhesion is approximately uniform across all ratio levels, validating the geometric generation process’s unbiased nature. Meanwhile, the distribution of actual adhesion aligns with the characteristics of genuine handwriting (i.e., lower adhesion ratios are more common), ensuring the dataset’s realism and ability to challenge segmentation algorithms.

**Table 2:** Distribution of adhesion degree (Unit: %).

Dataset	Adhesion type	0%	5%	10%	15%	20%	25%	30%
TDTS-TR	Rectangular adhesion	0.00	16.67	16.86	16.84	16.31	16.79	16.45
	Actual adhesion	63.67	11.64	9.47	7.19	4.48	2.44	1.11
TDTS-TS	Rectangular adhesion	0.00	16.57	16.89	16.72	16.25	16.92	16.65
	Actual adhesion	65.76	11.01	9.29	6.68	3.90	2.46	0.90

### 3.5 Chapter summary

This chapter constructs a standardized benchmark dataset (TDTS) for two-digit touching image segmentation. By designing a controllable geometric adhesion model, implementing a class-balanced sampling strategy, and adopting anti-confusion pixel fusion and label-encoding rules, all design objectives outlined in Section 3.1 are fully met. The resulting dataset features clear adhesion patterns, controlled variables, and balanced classes, effectively isolating biases introduced by the data. As such, it provides a fair and precise benchmark for evaluating the performance of various segmentation algorithms. In subsequent chapters, this dataset will serve as the foundation for systematic experimental validation of the “cutting domain”-based graph-theoretic segmentation framework proposed in Chapter Methods, which integrates threshold-free search-space construction with classical shortest-path algorithms.

## 4 Methods

### 4.1 Problem definition and unified evaluation framework

#### 4.1.1 Problem definition and evaluation protocol.

The objective of two-digit touching image segmentation is to partition an input image *I* containing two adhesive characters into two independent character regions ($I_{L}$ for the left character and $I_{R}$ for the right character) via a continuous segmentation path *P*, which serve as the input for the subsequent recognition modules simultaneously. To ensure fair and precise performance comparison among different segmentation algorithms, this study establishes a unified evaluation framework with three core specifications, as detailed below:

- **Core evaluation metric:** The overall recognition accuracy of the two segmented characters is adopted as the primary evaluation metric. This metric directly reflects the practical effectiveness of each segmentation algorithm, integrating both segmentation precision and compatibility with downstream recognition tasks.
- **Unified spatial constraint:** All segmentation operations are confined within a horizontal rectangular region spanning columns (x∈[13,27]). This constraint is derived by slightly expanding the actual potential fusion zone of characters (x∈[14,19]) defined during TDTS dataset construction (Chapter Materials). It delineates a consistent “search corridor with defined boundaries” for all algorithms, eliminating evaluation bias caused by arbitrary differences in the selection of segmentation start/end points.
- **Standardized evaluation pipeline:** To ensure that performance discrepancies are attributed solely to the inherent logic of segmentation algorithms, all methods follow the same pipeline.

This standardized framework ensures that subsequent performance comparisons objectively reflect the effectiveness and robustness of each segmentation algorithm’s core logic.

#### 4.1.2 Methodology classification and overall workflow.

To systematically address the touching digit segmentation problem, this chapter proposes and compares two primary technical paradigms, as illustrated in the overall framework (Fig 3). The Vertical Projection Method and the Constrained Watershed Algorithm represent traditional heuristic-based baselines, while U-Net represents a deep learning-based segmentation baseline; together, these three methods serve as the baselines for performance benchmarking. The proposed method consists of two sequential core steps: first, abstracting the preprocessed image into a structured cutting domain, which transforms the segmentation task into a graph-based optimal path search problem; second, applying path-finding algorithms on the graph constructed by the cutting domain—either Dijkstra’s algorithm for global optimality or a family of A* algorithms with directional biases and penetration mechanisms for heuristic search.

**Fig 3:**
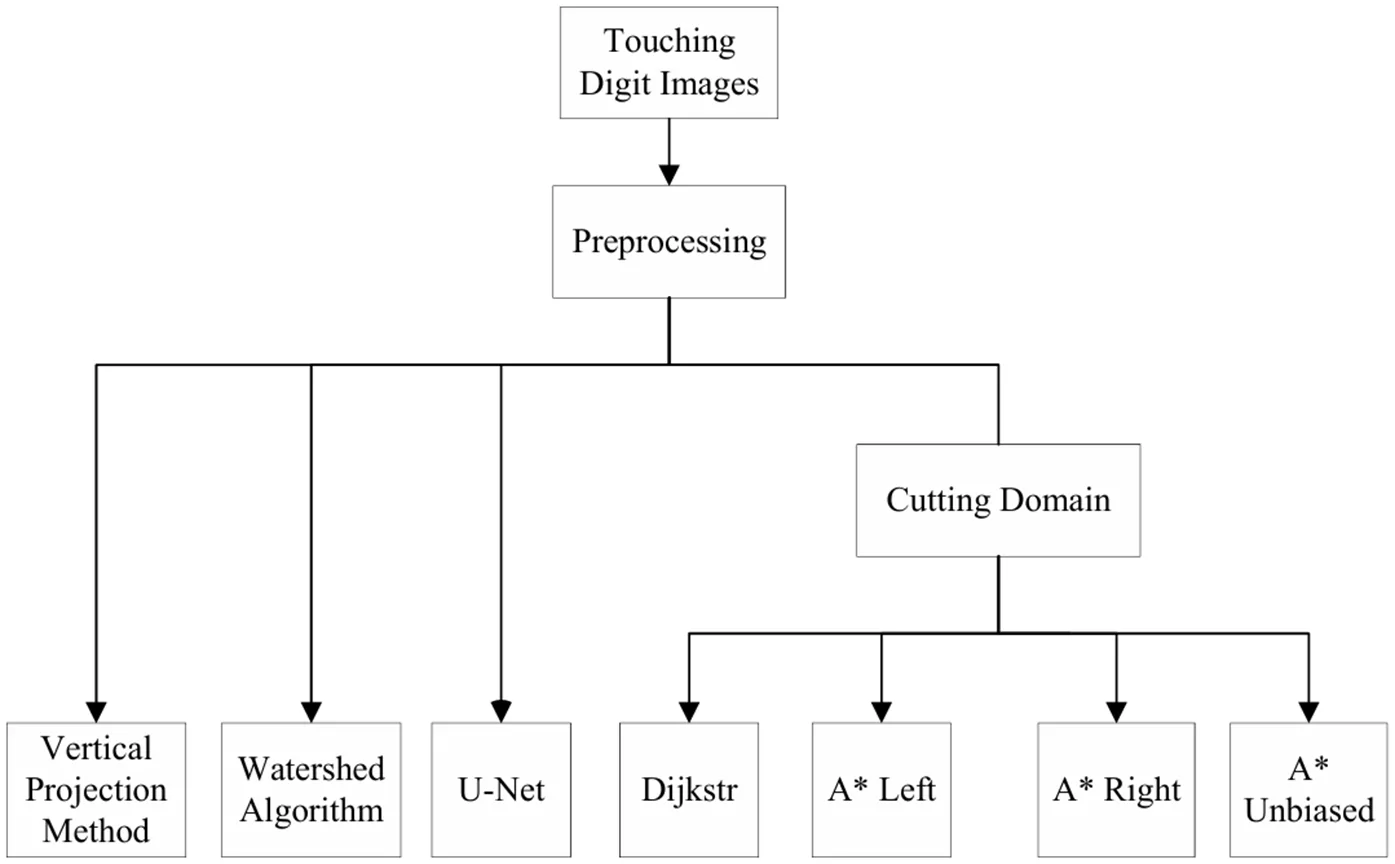
Comparative framework of key segmentation algorithms.

In this framework, the preprocessing stage primarily includes denoising and region delineation. The denoising algorithm is designed to eliminate interference from random scatter noise introduced during TDTS construction, thereby avoiding noise-induced segmentation errors. Based on the local 3σ criterion, the algorithm detects and replaces abnormal pixel values, effectively suppressing noise while preserving the integrity of character strokes. Region delineation specifies the lateral working range for all segmentation algorithms, ensuring consistent comparison conditions. Representative images before and after denoising are shown in Fig 4 and Fig 5, respectively.

**Fig 4:**
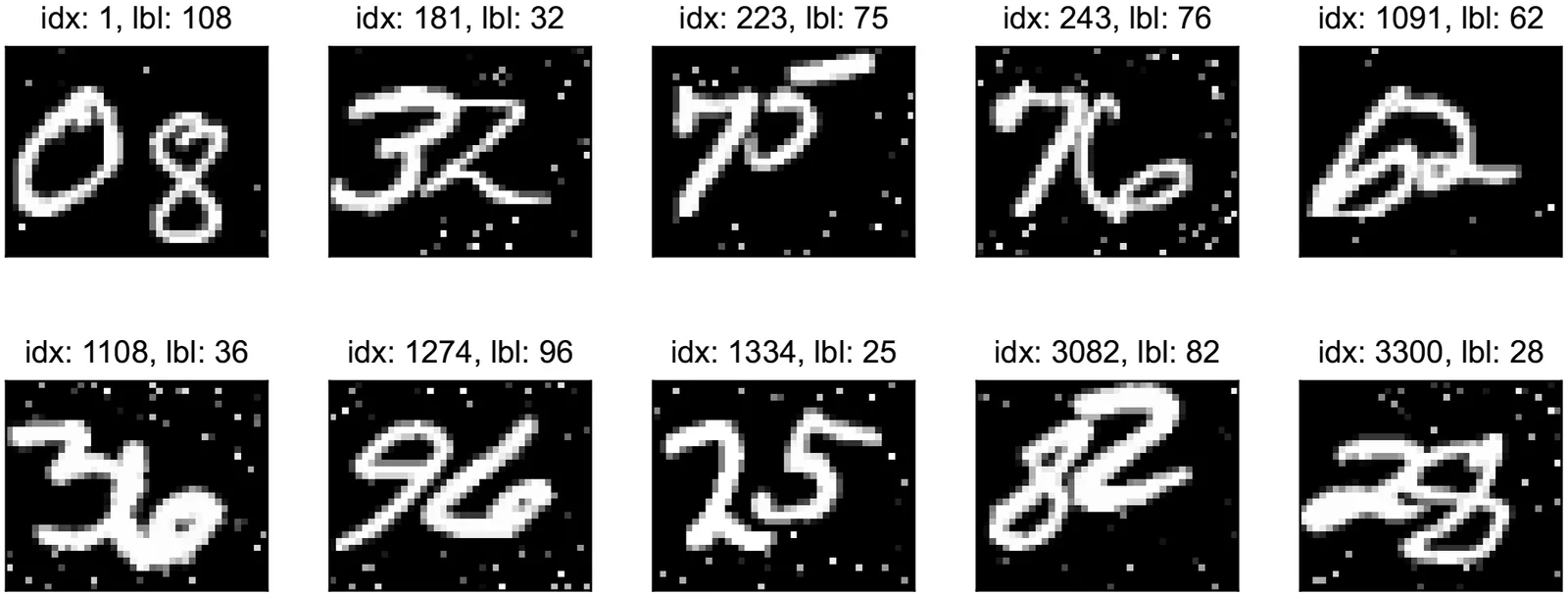
Representative original touching images (with scatter noise).

**Fig 5:**
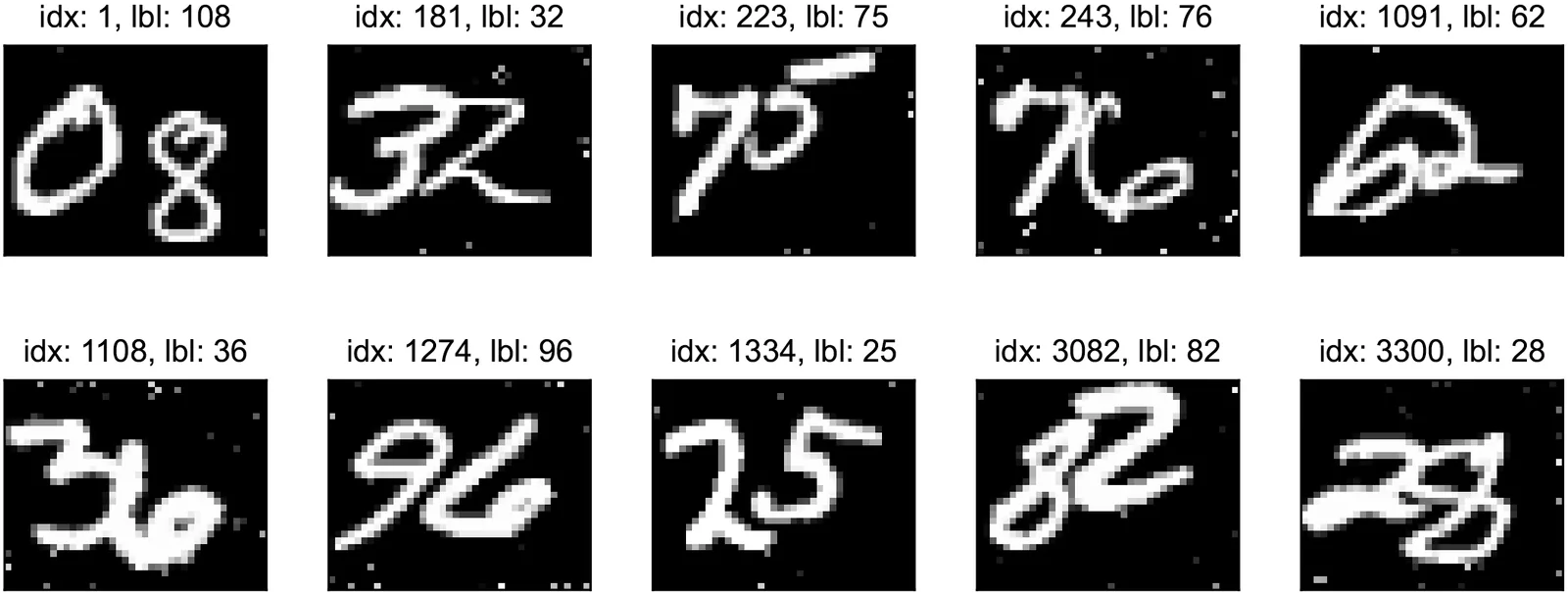
Representative denoised touching images.

As shown in the figures, the denoising algorithm effectively eliminates scattered noise without distorting character structures, thereby reducing noise interference in subsequent segmentation and recognition tasks.

### 4.2 Cutting domain: Construction of a structured search space

#### 4.2.1 Design philosophy and problem transformation.

The core challenge in digit touching segmentation is identifying a continuous, reasonable separation boundary between two adhesive characters. Traditional methods operate directly in the pixel space, facing inherent limitations such as a vast search space and susceptibility to interference from local grayscale fluctuations and residual noise. To address these issues, this study proposes a paradigm transformation: reframing the image segmentation problem as an optimal path search problem in graph theory [42].

To realize this transformation, a discrete graph structure—the cutting domain—is constructed, adhering to three core design principles to provide an optimized, controllable input for path-finding algorithms:

- **Spatial constraint principle:** The search scope is strictly confined to the predefined corridor (x∈[13,27]), excluding the blank areas on both sides of the characters to reduce redundant search and improve efficiency.
- **Topological connectivity principle:** The generated cutting domain must ensure that there is at least one connected path within its point set, with the start point on the image’s top boundary and the end point on its bottom boundary. This design shifts the segmentation problem from “whether a feasible segmentation path exists” to “which path is optimal,” laying the foundation for implementing path-search algorithms.
- **Cost-guided principle:** Image grayscale values are mapped to path traversal costs, with low-cost paths prioritizing background regions (pixel value = 0) or faint strokes. This ensures that the optimal path on the graph corresponds to a segmentation line that minimally damages character structural integrity.

Through the construction of the cutting domain, the original image *I* is converted into a traversable region mask, which can be modeled as an implicitly connected graph *G*(*V*, *E*). Here, the vertex set *V* consists of all traversable pixel coordinates in the mask, and the edge set *E* is defined by 8-neighborhood connectivity (i.e., an edge exists between adjacent traversable pixels). Thus, the segmentation task is precisely mapped to finding the optimal path on graph *G* from the top traversable vertex set *V*_top_ to the bottom traversable vertex set *V*_bottom_.

#### 4.2.2 Construction algorithm and implementation.

The cutting domain is specifically constructed using a cost-priority-based region growing strategy, whose workflow is illustrated in Fig 6.

**Fig 6:**
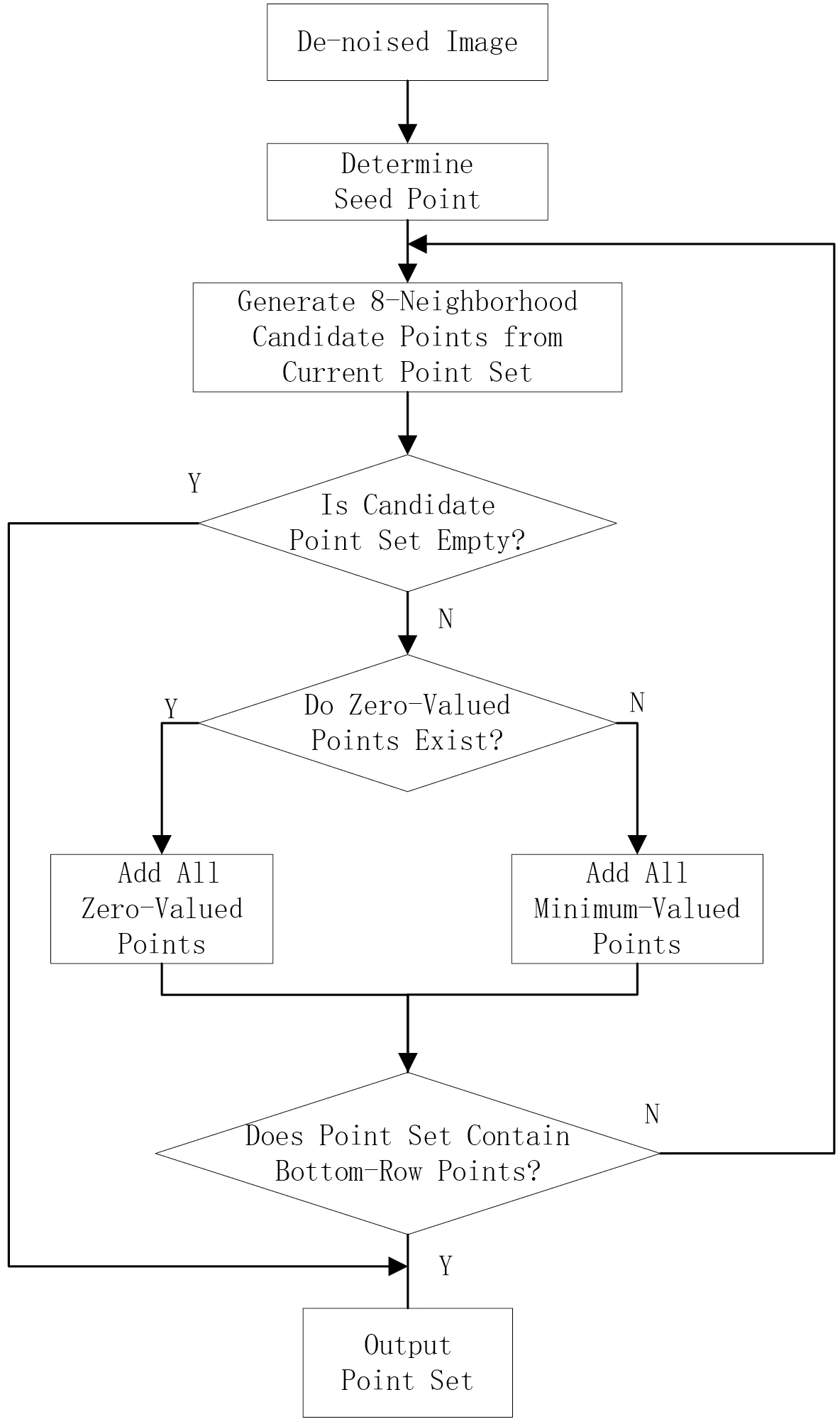
Flowchart of the cutting domain construction algorithm.

The core of this algorithm simulates a “fluid seepage” process within the constrained region [43,44]: starting from the top boundary, it spreads downward along the path of least resistance (lowest pixel cost), following these key steps:

- **Seed initialization:** The pixel with the minimum grayscale value in the top row (*y* = 0) of the constrained region is selected as the initial growth seed, ensuring the growth process starts from the lowest-cost region.
- **Iterative region growing:** Starting from the current traversable region, 8-neighborhood candidate pixels are inspected. Candidates are considered in a priority order: first, pure background pixels (grayscale value = 0); if none exist, the candidate with the minimum grayscale value is selected. A priority queue manages this process, ensuring expansion always proceeds from the current lowest-cost frontier.
- **Termination condition:** The growth process stops when the newly incorporated pixels include a point from the bottom row (*y* = 31), or when no new valid candidates can be added. This guarantees that the generated cutting domain contains at least one vertically connected path, satisfying the topological connectivity requirement.

To make explicit the cost function underlying this growth process, let *I*(*p*) denote the grayscale value of the denoised image at pixel *p* = (*x*,*y*), with I(p)∈[0,255] and *I*(*p*) = 0 corresponding to pure background. For a pixel *p*, let *N*_8_(*p*) denote its 8-neighborhood, extended to any pixel set *D* by $N_{8}(D):={⋃}_{p\in D}N_{8}(p)$. The growth priority cost is defined as:


$$\begin{array}{c} C_{\text{domain}}(p)=I(p). \end{array}$$ (2)

Region growing proceeds as a priority-queue-driven best-first search: starting from the seed domain *D*_0_ (Seed Initialization), at each step t=0,1,2,..., among the boundary candidate set $N_{8}(D_{t})⧵D_{t}$, the pixel


$$\begin{array}{c} p_{t}^{*}=\underset{p\;\in \;N_{8}(D_{t})⧵D_{t}}{\arg\;\min}C_{\text{domain}}(p) \end{array}$$ (3)

is selected and merged into the domain, $D_{t+1}=D_{t}\cup \{p_{t}^{*}\}$, until the bottom boundary *V*_bottom_ is reached at some terminal step *T* or no valid candidate remains (Termination Condition). Since pure background pixels satisfy *I*(*p*) = 0, they are naturally assigned the highest priority under this cost definition, which is exactly equivalent to the rule stated above (pure background pixels first, otherwise the candidate with the minimum grayscale value). The output of this growth process constitutes the graph model:


$$\begin{array}{c} V:=D_{T},\;E:=\{(p,q)\in V\times V:q\in N_{8}(p\}. \end{array}$$ (4)

As illustrated in Fig 7, this threshold-free algorithm can automatically isolate the touching region and generate a connected set of low-cost cutting points. By constructing this structured search space, it successfully decouples the two core processes of adhesion region localization and segmentation path optimization, thereby addressing the limitations of traditional coupled methods.

**Fig 7:**
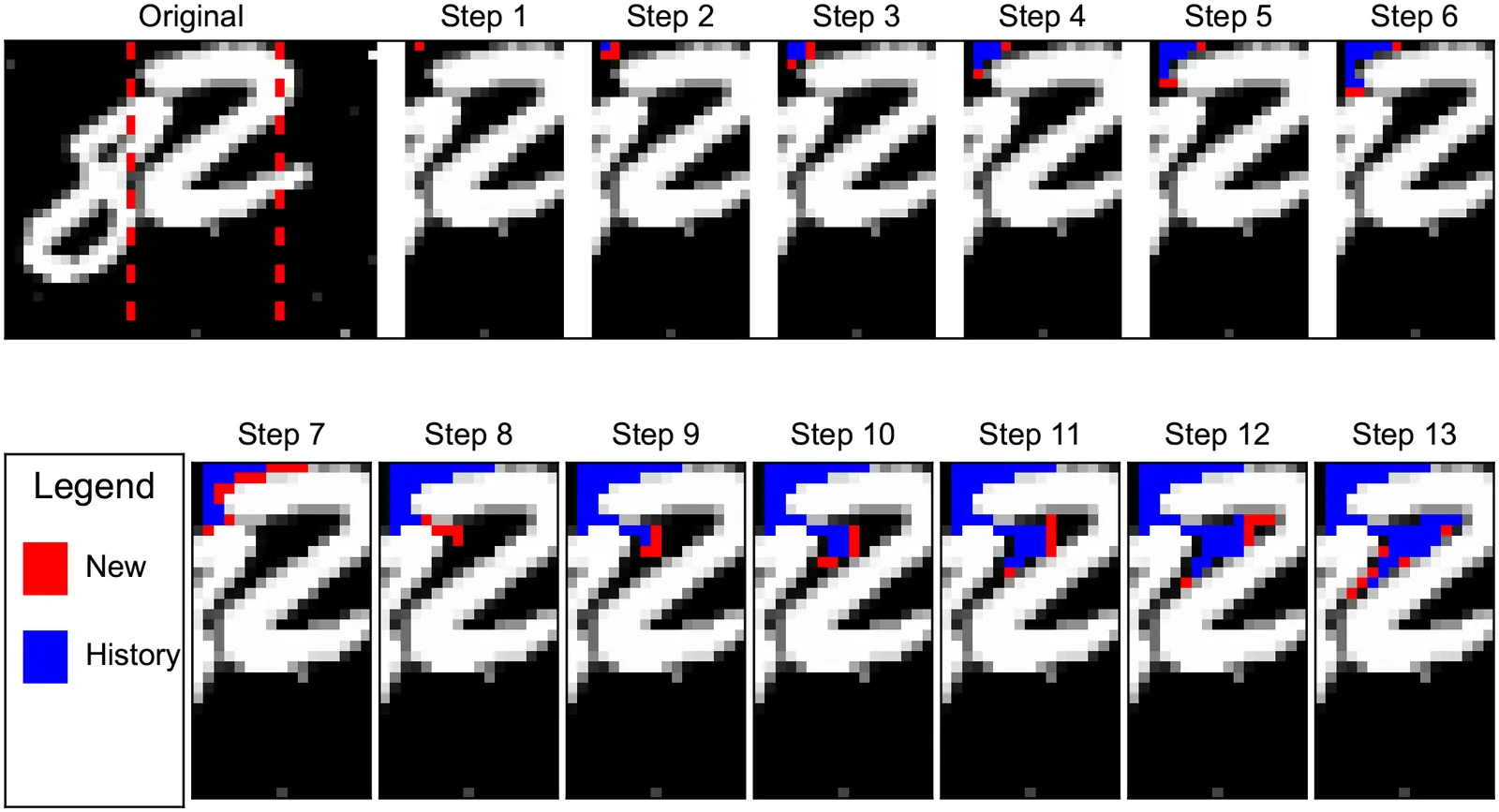
Schematic illustration of key stages in the cutting domain growth process.

### 4.3 Path search algorithms based on the cutting domain

On the graph model *G*(*V*, *E*) of the cutting domain, the segmentation task is transformed into finding the optimal path from *V*_top_ to *V*_bottom_. This section first introduces the unified foundational model shared by all path-search algorithms, followed by detailed descriptions of Dijkstra’s algorithm (for global optimality) and the A* algorithm family (with directional guidance and intelligent penetration capabilities).

#### 4.3.1 Unified foundational model and shared characteristics.

All path-search algorithms in this study share the following foundational design and characteristics to ensure consistency and comparability:

1. **Consistent graph input:** All algorithms operate on the cutting domain graph *G*, ensuring a consistent input basis.
2. **Multi-source, multi-sink model:** The source set comprises all traversable pixels in the top boundary of the cutting domain, and the sink set comprises all traversable pixels in the bottom boundary. This simultaneous search from all potential sources avoids local optimality traps associated with a single starting point.
3. **Base traversal cost:** Movement in an 8-neighborhood uses a uniform unit step cost (a chessboard/Chebyshev-style distance), independent of direction:

$$\begin{array}{c} d(u,v)=1,\;\forall v\in N_{8}(u), \end{array}$$ (5)

where *N*_8_(*u*) is as defined in Section 4.2.2. This unit step cost marks only whether a move has occurred; it does not itself encode directional anisotropy, which is instead carried explicitly and separately by the multiplicative bias coefficients below, so as to avoid double-encoding directional preference.

4. **Directional bias coefficients:** To encourage path consistency, a multiplicative bias coefficient β(u,v) is applied to the A* variants. In the vertical direction, moves along the primary growth direction carry a coefficient $\beta_{\text{vert}}=0.8$, while moves against it are penalized with $\beta_{\text{vert}}=1.5$; for the Left-/Right-Biased A* variants, an additional horizontal bias is applied, with $\beta_{\text{horz}}=0.9$ for moves along the bias direction and $\beta_{\text{horz}}=1.1$ for moves against it, while Unbiased A* takes $\beta_{\text{horz}}=1$. Dijkstra’s search applies no directional bias in either direction ($\beta_{\text{vert}}=\beta_{\text{horz}}=1$). The complete traversal cost for an edge (u,v)∈E is therefore:

$$\begin{array}{c} w(u,v)=d(u,v)\cdot \beta_{\text{vert}}(u,v)\cdot \beta_{\text{horz}}(u,v). \end{array}$$ (6)

The Conditional Penetration Mechanism (Section 4.3.3) constitutes an exception branch to Eq 6: when its trigger condition is met and the estimated detour cost exceeds the threshold, the jump edge (current,pierce_point) is instead assigned a fixed cost of 9 (Unbiased A*) or 4 (Left-/Right-Biased A*), replacing rather than multiplying Eq 6; this branch is not used by Dijkstra’s search.

5. **Robustness mechanisms:** Anomaly detection truncates cyclical or excessively long paths. A predefined fallback scheme (e.g., returning a central vertical line at *x* = 20) guarantees that a valid segmentation path is always returned, ensuring pipeline robustness.

It should be noted that grayscale intensity does not appear as a multiplicative or additive term in the edge cost *w*(*u*,*v*) defined in Eq 6: grayscale information has already been fully absorbed at the cutting-domain construction stage (Section 4.2.2, Eq 2), where only pixels satisfying the low-grayscale/background-priority growth rule are admitted into the vertex set *V*. Consequently, the path-search cost defined in this section operates purely on step count and directional consistency within an already grayscale-filtered search space, without re-weighting grayscale information a second time.

#### 4.3.2 Dijkstra’s global optimal path search.

Dijkstra’s algorithm is employed to find the globally shortest path (i.e., the path with the minimum accumulated cost) from the source set to the sink set within the cutting domain. Its primary advantage lies in guaranteeing a theoretically optimal solution under the defined cost function, serving as a performance upper bound benchmark for the cutting domain framework.

#### 4.3.3 The A* heuristic search family and conditional penetration mechanism.

To explore the effectiveness of different search strategies and handle complex adhesion cases (e.g., pixel-level penetration between characters), this study implements three variants of the A* algorithm: Left-Biased A*, Right-Biased A*, and Unbiased A*. Since inherent writing habits in contiguous digit pairs often yield highly asymmetric adhesion morphologies, explicitly tailoring directional biases and hyperparameters to the domain’s asymmetry is crucial for optimizing algorithm performance and convergence [45]. The core differences lie in the differentiated setting of horizontal movement cost coefficients and biases in start/end point selection, which guide the algorithm to prioritize exploration in specific directions to adapt to asymmetric adhesion morphologies [46].

The Conditional Penetration Mechanism is a key innovation of the A* algorithm family, designed to address narrow pixel-level adhesion between characters. It adheres to the principle of “detour first, reasonable penetration” to balance path continuity and character integrity:

- **Trigger condition:** Penetration is only considered when character strokes entirely block the current pixel’s front and adjacent regions, and a reconnectable traversable region exists within a predefined distance (set to 5 pixels in this study).
- **Cost trade-off:** When the trigger condition is met, the algorithm first calculates the estimated detour cost. Penetration is executed only if the detour cost exceeds a predefined threshold (7.2). A fixed penetration cost is then assigned: 9 for Unbiased A*, and a lower cost of 4 for the Left- and Right-Biased variants. This economic trade-off ensures path continuity while minimizing damage to character structures.

The penetration mechanism enhances the algorithm’s adaptability to extreme adhesion cases, while the directional bias strategies provide flexibility for handling asymmetric adhesion patterns.

### 4.4 Brief description of baseline methods

To provide comparison baselines, this chapter implements two classic traditional segmentation methods and one modern supervised segmentation network under the same unified evaluation framework (spatial constraint, denoising, and pipeline):

- **Vertical projection method:** Within the constrained region (x∈[13,27]), the vertical projection (sum of grayscale values per column) is calculated. The column with the minimum projection value is selected as the segmentation line. This method is simple and computationally efficient but relies solely on global statistical information, making it sensitive to local adhesion and residual noise—limitations visualized in Fig 8.
- **Constrained watershed algorithm:** A distance transform is applied to the denoised image. Within the constrained region, local minima are detected as markers to guide the watershed algorithm in generating segmentation boundaries. This method adapts better to character contour shapes than the vertical projection method, but is sensitive to marker selection and parameters, and prone to over-segmentation under severe adhesion. Visualization results are shown in Fig 9.
- **U-Net supervised segmentation baseline:** To contrast supervised and unsupervised segmentation paradigms within the same framework, a compact U-Net is implemented. Ground-truth training masks are automatically derived from the character-placement coordinates and pixel-fusion rule used during TDTS generation, labeling each pixel into one of three classes (background, left character, or right character). At evaluation, U-Net outputs a per-pixel three-class prediction for the entire touching image; for each class, the original grayscale is retained at pixels predicted as that class and zeroed elsewhere, yielding two single-character images that are fed into the same recognition model. When the trained weights are transferred to out-of-domain data, no fine-tuning is performed, so as to assess the generalization boundary of supervised segmentation under domain shift.

**Fig 8:**
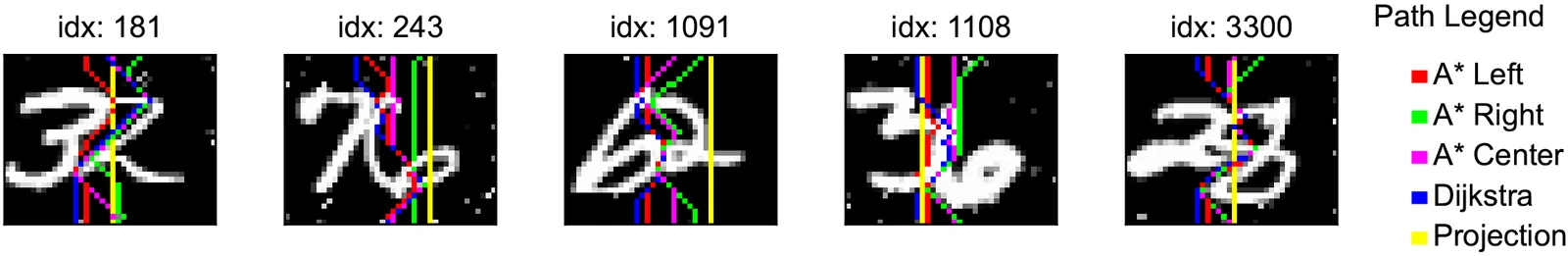
Comparison of segmentation results by different algorithms on representative TDTS samples.

**Fig 9:**
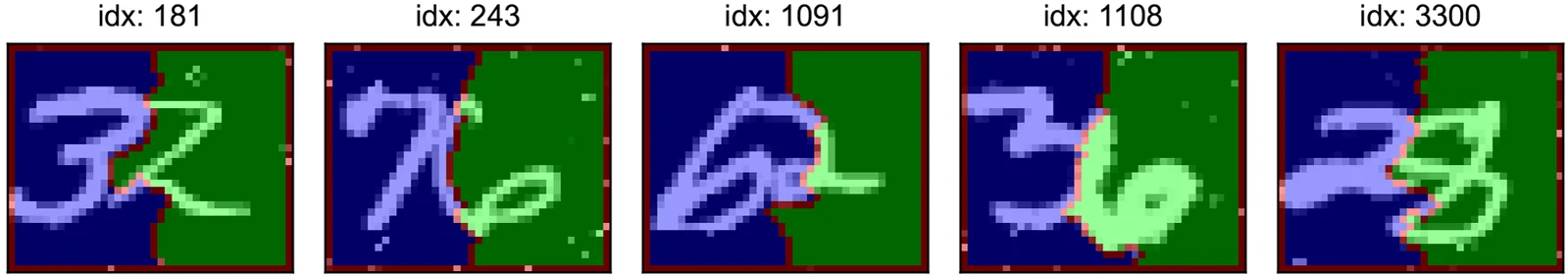
Visualization of the constrained watershed algorithm results.

These three methods represent typical implementations of the “segment-then-recognize” paradigm. Their performance and inherent limitations will be quantitatively analyzed and compared with the proposed method in the Chapter Experiment.

### 4.5 Chapter summary

This chapter systematically proposes a “cutting domain”-based modeling framework and corresponding path-search algorithms for touching handwritten digit segmentation. The core contributions are summarized as follows:

- **Structured search space construction:** A cutting domain construction method that transforms image segmentation into a graph-based optimal path search problem, decoupling the task into independent modeling and solution stages.
- **Unified path-search model:** A foundational model based on the cutting domain graph, implementing both Dijkstra’s algorithm and A* heuristic variants for solutions ranging from global optimality to targeted search.
- **Comprehensive baseline implementation:** Under the same spatial constraints and evaluation framework, comparison baselines spanning traditional heuristic-based methods (Vertical Projection Method, Constrained Watershed Algorithm) and a deep supervised method (U-Net) are implemented, providing a fair basis for cross-paradigm performance evaluation.

This chapter provides a comprehensive, innovative, and comparable methodological framework for handwritten digit segmentation. The Chapter Experiment will conduct systematic experimental validation and in-depth comparative analysis of these methods using the standardized TDTS dataset constructed in Chapter Materials, thereby verifying the superiority of the proposed framework.

## 5 Experiment

### 5.1 Experimental setup and evaluation framework

This chapter aims to systematically validate the graph-theoretic segmentation algorithm based on the cutting domain proposed in Chapter 4. Experiments are conducted using the standardized TDTS dataset constructed in Chapter 3, which includes the training-source synthetic library (TDTS-TR) and the test-source synthetic library (TDTS-TS). All tests are performed under a unified hardware and software environment (Python 3.9, PyTorch 1.12.0, Intel i7-3630QM CPU, 16GB RAM) to ensure the reproducibility of experimental results.

To guarantee fair comparison among algorithms, this study establishes a rigorous evaluation process as illustrated in Fig 10. This pipeline strictly adheres to the unified evaluation specifications defined in Chapter 4 (e.g., fixed spatial constraint region x∈[13,27], standardized preprocessing), ensuring that performance discrepancies are exclusively attributed to the inherent logic of segmentation algorithms rather than inconsistencies in the evaluation environment.

**Fig 10:**
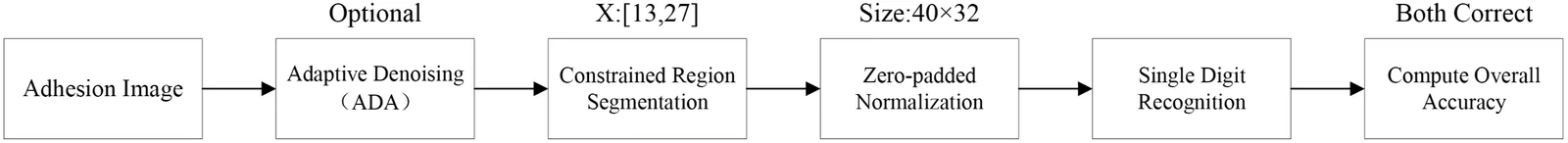
Evaluation pipeline for touching handwritten digit segmentation and recognition.

The remainder of this chapter is organized as follows: Section 5.2 presents the core recognition results and analyzes the key factors underlying algorithm performance; Section 5.3 validates the framework’s computational efficiency, robustness, and design principles; Section 5.4 further evaluates the framework’s generalization to real-world handwritten digit strings; Section 5.5 presents a cross-paradigm comparison against a deep supervised segmentation baseline (U-Net); and Section 5.6 summarizes the chapter’s main findings.

### 5.2 Core results and comprehensive analysis

#### 5.2.1 Recognition model selection and overall algorithm performance.

To establish a robust evaluation benchmark compatible with segmentation-derived noise, three single-character recognition models are trained, with their details and performance summarized in Table 3. The characteristics of each model are as follows:

- **Clean recognition model (CRM):** The images for the training and test sets are derived from the compact MNIST characters (Directory Data3 in Chapter Materials) using zero-padding normalization.
- **Noisy recognition model (NRM):** The images for the training and test sets are generated by first adding random noise to the normalized canvas, then overlaying the compact MNIST characters on it.
- **Mixed recognition model (MRM):** Its training set is formed by merging the complete training sets of the aforementioned CRM and NRM, and its test set is likewise constructed by merging their complete test sets.

**Table 3:** Training and test accuracy of single-character recognition models (unit: %).

Models	TDTS-TR	TDTS-TS
CRM	98.83	98.16
NRM	98.01	97.95
MRM	99.36	98.03

Note: TDTS-TR: Train-derived digit touching set; TDTS-TS: Test-derived digit touching Set.

However, the results in Table 3 reflect model performance under ideal controlled conditions. In the complete segmentation-recognition pipeline (Chapter Methods), the input to the recognition module consists of single-character images that have undergone denoising, segmentation, and normalization—images that contain “segmentation-induced noise” with a distribution distinct from that of the test sets in Table 3. Thus, empirical testing across the entire pipeline is essential to selecting the optimal recognition model.

Each of the three models is integrated into the unified pipeline and tested across six segmentation algorithms.

The overall recognition accuracy is summarized in Table 4, with core conclusions drawn as follows:

- **MRM outperforms other models:** Across all segmentation algorithms and test sets, the performance ranking of recognition models is consistently MRM > CRM > NRM. This confirms that the MRM, trained on mixed-noise data, exhibits the best robustness to compound noise generated by the segmentation pipeline. Thus, MRM is adopted as the fixed recognition benchmark for all subsequent experiments.
- **Graph-theoretic methods surpass traditional baselines:** Cutting domain-based graph-theoretic algorithms (Dijkstra, A* variants) significantly and stably outperform traditional methods (VPM, WA). Among them, the Left-Biased A* algorithm achieves the highest accuracy of 93.13% on TDTS-TS, demonstrating robust near-optimal performance.
- **Algorithm ranking aligns with dataset characteristics:** Left-Biased A* outperforms other A* variants, which correlates with the prevalent left-leaning adhesion morphology in the TDTS dataset (Chapter 3). This validates the effectiveness of the directional bias strategy designed in Chapter Methods.
- **Strong generalization ability verified:** Despite TDTS-TS having more challenging statistical characteristics (CV: 0.1110 > 0.0631), graph-theoretic methods maintain comparable or slightly better performance on TDTS-TS than on TDTS-TR. This is attributed to the higher proportion of “actual non-adhesion” simple samples in TDTS-TS (65.76% > 63.67%, Chapter Materials), which are easier to process. This indirectly confirms the algorithms’ strong generalization ability—performance does not degrade in mixed environments with complex distributions and simple samples.

**Table 4:** Recognition accuracy of different recognition models combined with segmentation algorithms (unit: %).

Recognition models	Test set	Left-biased A*	Right-biased A*	Unbiased A*	Dijkstra	VPM	WA
MRM	TDTS-TR	92.67	90.47	92.15	92.25	87.92	82.89
	TDTS-TS	93.13	90.77	92.68	92.63	88.21	82.34
CRM	TDTS-TR	92.14	89.84	91.64	91.79	87.05	82.22
	TDTS-TS	92.68	90.26	92.12	92.22	87.59	82.46
NRM	TDTS-TR	91.62	89.33	91.12	91.24	86.57	80.78
	TDTS-TS	92.00	89.66	91.49	91.48	86.83	81.02

Note: VPM = Vertical projection method; WA = Constrained watershed algorithm; TDTS-TR: Train-derived digit touching set; TDTS-TS: Test-derived digit touching set.

#### 5.2.2 Analysis of the denoising module’s contribution.

To quantify the effect of the adaptive denoising algorithm proposed in chapter methods, an ablation experiment is conducted by comparing performance with and without the denoising module enabled. Performance change is defined as $\Delta =Acc_{\text{exp}}-Acc_{\text{full}}$, where *Acc*_exp_ is accuracy without denoising and *Acc*_full_ is accuracy with denoising. Results are summarized in Table 5.

**Table 5:** Accuracy in the denoising module ablation experiment (unit: %).

Segmentation algorithm	TDTS-TR	TDTS-TR Δ	TDTS-TS	TDTS-TS Δ	Observation
Left-Biased A*	92.73	−0.05	93.01	+0.12	Slight fluctuation
Right-Biased A*	90.48	−0.01	90.64	+0.13	Slight fluctuation
Unbiased A*	92.16	0.00	92.51	+0.17	Slight fluctuation
Dijkstra	92.30	−0.04	92.54	+0.09	Slight fluctuation
VPM	87.87	−0.05	88.08	−0.13	Slight decrease
WA	82.68	−0.21	82.09	−0.25	Slight decrease

Note: VPM = Vertical projection method; WA = Constrained watershed algorithm; TDTS-TR: Train-derived digit touching set; TDTS-TS: Test-derived digit touching set.

The denoising module’s performance impact is generally limited, primarily because the MRM already exhibits strong noise robustness. Thus, ADA serves as an optional preprocessing step that enhances the framework’s completeness in extreme-noise scenarios rather than a mandatory component.

#### 5.2.3 Analysis of key factors in cutting domain construction.

The cutting domain is the foundational component of the proposed framework, with topological connectivity constraints and neighborhood selection as its core design elements (Chapter methods). Ablation experiments are conducted to quantify their impacts.

- **Necessity of connectivity constraints:** To verify the value of spatial connectivity constraints, the constraint is removed (only the minimum grayscale value per row is considered). Results in Table 6 show that most algorithms experience significant performance degradation, confirming that connectivity constraints are essential for effective segmentation.

**Table 6:** Accuracy changes with modifications to connectivity constraints (unit: %).

Segmentation algorithm	TDTS-TR	TDTS-TR Δ	TDTS-TS	TDTS-TS Δ	Performance note
Left-Biased A*	93.25	**+0.58**	93.54	**+0.41**	Slight increase
Right-Biased A*	78.11	**−12.36**	78.02	**−12.75**	Sharp drop
Unbiased A*	89.22	**−2.94**	89.51	**−3.17**	Significant drop
Dijkstra	89.90	**−3.36**	89.27	**−3.36**	Significant drop

Note: TDTS-TR: Train-derived digit touching set; TDTS-TS: Test-derived digit touching set. $\Delta =Acc_{\text{exp}}-Acc_{\text{full}}$

Notably, Left-Biased A* exhibits a slight accuracy increase, which is attributable to two implementation-level asymmetries rather than dataset adhesion alone. First, the horizontal bias coefficients (0.9/1.1) in the A* cost function consistently reward movement toward the left portion of the search corridor, which is itself left-of-center relative to the character fusion zone; when connectivity is removed and only the minimum grayscale value per row is retained, the resulting mask is more likely to be disconnected, and this bias reduces the frequency with which Left-Biased A* falls back to the forced straight-line path under such conditions. Second, Right-Biased A* is more frequently pushed toward the corridor’s right side, where this row-wise mask is less likely to offer a reconnection point within the pierce-search window, triggering the forced-path fallback that can cut directly through character strokes. This explains the sharp asymmetry in Table 6 without contradicting the necessity of connectivity constraints in realistic, adhesion-location-unknown deployment.

- **4-Neighborhood vs. 8-Neighborhood comparison:** Replacing 8-neighborhood growth (Chapter 4) with 4-neighborhood growth yields slight performance improvements for most algorithms (Table 7). For this phenomenon, we attribute it to the vertical morphology of the character fusion zone, which is highly compatible with 4-neighbor connectivity and thus better aligns with the practical technical requirements for character segmentation.

**Table 7:** Accuracy comparison: 4-neighborhood versus 8-neighborhood growth (unit: %).

Segmentation algorithm	TDTS-TR	TDTS-TR Δ	TDTS-TS	TDTS-TS Δ	Performance note
Left-Biased A*	92.28	−0.39	92.77	−0.36	Slight decrease
Right-Biased A*	91.30	+0.83	91.68	+0.91	Slight increase
Unbiased A*	92.17	+0.02	92.63	+0.05	Slight increase
Dijkstra	92.33	+0.08	92.80	+0.17	Slight increase

Note: TDTS-TR: Train-derived digit touching set; TDTS-TS: Test-derived digit touching set. $\Delta =Acc_{\text{exp}}-Acc_{\text{full}}$

### 5.3 Validation of algorithm efficiency, robustness, and design principles

This section evaluates the practical value of the graph-theoretic framework from three dimensions: computational efficiency, resource consumption, and task success rate. Results are presented in Table 8.

**Table 8:** Performance evaluation of graph-theoretic segmentation algorithms (on 10,000 test images).

Algorithm	Runtime (s)	Memory usage (MB)	No. of path cycles	Success rate
Left-Biased A*	10.22	24.34	17	100%
Unbiased A*	9.09	24.40	21	100%
Dijkstra	32.29	24.28	0	100%

Note: Runtime measured in seconds (s); Memory usage in megabytes (MB); No. of Path cycles indicates the number of detected cyclic paths; Success rate indicates the percentage of successfully segmented images out of 10,000 test images.

- **Balance between efficiency and accuracy:** The A* algorithm family (Left-Biased, Unbiased) runs 3–4 times faster than Dijkstra’s algorithm, with minimal accuracy loss. This provides flexible options for practical deployment: Dijkstra’s algorithm for ultimate accuracy, and A* variants for efficient real-time applications.To assess scalability to higher-resolution document images, we repeated the efficiency evaluation at 2×, 4×, and 8× the original resolution (80×64, 160×128, and 320×256; Table 9), using 1,000 samples per resolution on the same hardware as the primary experiments. Runtime for all three methods grows approximately in proportion to pixel count: each resolution doubling (a 4× increase in pixel count) corresponds to a 3.3–4.3× increase in runtime for Dijkstra and Unbiased A*, consistent with the expected complexity of graph search over a corridor whose size scales with image area. Notably, Unbiased A*’s efficiency advantage over Dijkstra widens at higher resolutions, from 3.45× at baseline to 4.82× at 8× resolution, whereas Left-Biased A*’s advantage narrows from 2.94× to 1.73×. We attribute this asymmetry to the Conditional Penetration Mechanism (Section 4.3.3): its bypass-search and reconnection windows scale with image resolution, and because Left-Biased A* triggers penetration more frequently than Unbiased A* (Section 5.2.3), each triggered event becomes proportionally more expensive at larger scales. This suggests Unbiased A* offers the most favorable efficiency-accuracy trade-off for high-resolution deployment, while a resolution-adaptive pierce-search window is a natural direction for future optimization.
- **Robustness guarantee:** All algorithms achieve a 100% task success rate. Dijkstra’s algorithm has zero path cycles, demonstrating the highest robustness. Although A* algorithms occasionally exhibit cycles (< 0.3%), their built-in fallback mechanisms (e.g., returning the central vertical line *x* = 20, Chapter Methods) ensure valid segmentation paths are always output, guaranteeing pipeline stability.

**Table 9:** Runtime and memory scaling with image resolution.

Resolution	Algorithm	Runtime (s)	Memory (MB)	Avg. Iterations	N
40×32 (baseline)	Dijkstra	0.00361 ± 0.00076	0.0329 ± 0.0110	318.9 ± 48.1	1000
	Unbiased A*	0.00105 ± 0.00088	0.0203 ± 0.0061	56.0 ± 50.9	1000
	Left-Biased A*	0.00123 ± 0.00092	0.0200 ± 0.0047	64.9 ± 52.6	1000
80×64 (2×)	Dijkstra	0.01508 ± 0.00300	0.2121 ± 0.0685	1280.4 ± 191.7	1000
	Unbiased A*	0.00348 ± 0.00438	0.0475 ± 0.0215	190.1 ± 255.3	1000
	Left-Biased A*	0.00582 ± 0.00642	0.0489 ± 0.0192	313.6 ± 359.8	1000
160×128 (4×)	Dijkstra	0.06420 ± 0.01228	1.1642 ± 0.2732	5177.0 ± 768.0	1000
	Unbiased A*	0.01371 ± 0.02075	0.1359 ± 0.1142	737.6 ± 1137.9	1000
	Left-Biased A*	0.03142 ± 0.03922	0.1538 ± 0.1033	1627.0 ± 2094.1	1000
320×256 (8×)	Dijkstra	0.24995 ± 0.03967	5.0628 ± 1.2252	20824.3 ± 3073.8	1000
	Unbiased A*	0.05185 ± 0.08087	0.4351 ± 0.4869	2958.9 ± 4738.1	1000
	Left-Biased A*	0.14456 ± 0.16200	0.5878 ± 0.4808	7588.9 ± 8913.7	1000

*Note*: Values are reported as mean ± standard deviation over *N* = 1000 samples per resolution. Runtime in seconds, memory in megabytes. Resolution multiples (2×, 4×, 8×) are relative to the baseline 40×32.

### 5.4 Evaluation on real-world handwritten digit strings

#### 5.4.1 CVL dataset.

To assess generalization beyond the synthetic TDTS benchmark, we additionally evaluate our framework on the CVL (Computer vision Lab) handwritten digit database [47], whose touching patterns arise from natural handwriting rather than controlled synthesis. Two-digit candidates were automatically generated from CVL digit strings: vertical projection first located inter-character valleys as cut boundaries, yielding *N* character blocks that were then combined into N−1 adjacent two-digit candidates. A final manual pass retained genuinely touching samples, with at least 15 samples retained per class. A recognition model, structurally identical to CRM/NRM/MRM, was retrained on the CVL Single Digit subset to avoid conflating recognition-domain shift with segmentation quality. Dataset statistics are summarized in Table 10.

**Table 10:** CVL evaluation dataset and recognition baseline.

Item	Value
Two-digit samples	1,953
Classes covered	75
Single-char training set	7,000
Single-char test set	21,780
Single-char train accuracy (%)	98.56
Single-char test accuracy (%)	97.06

#### 5.4.2 Evaluation setup and results.

Since CVL samples lack the generation-time adhesion coordinates available for TDTS, the cutting-domain corridor is instead centered per image at the midpoint between the content’s geometric center and its projection-weighted centroid:


$$\begin{array}{c} c_{\text{center}}=0.5\times \left(c_{\text{geo}}+c_{\text{mass}}\right), \end{array}$$ (7)

where *c*_geo_ is the geometric center of the content bounding box and *c*_mass_ is its projection-weighted centroid, with the corridor radius proportional to content width (margin_frac=0.15, selected via grid search). All other pipeline components (denoising, cost function, recognition protocol) are unchanged from Chapter Methods. Following the algorithm subset evaluated in Table 4, we evaluate Left-Biased A*, Right-Biased A*, Unbiased A*, Dijkstra, and VPM; a sample is counted correct only if both digits are recognized correctly. Results are shown in Table 11.

**Table 11:** End-to-end recognition accuracy on CVL (*N* = 1,953) versus TDTS-TS (Table 4).

Method	CVL Acc.	TDTS-TS Acc.	Δ
Left-Biased A*	89.7	93.13	−3.4
Right-Biased A*	90.0	90.77	−0.8
Unbiased A*	91.4	92.68	−1.3
Dijkstra	90.3	92.63	−2.3
VPM	84.4	88.21	−3.8

Note: Accuracy in %; $\Delta =Acc_{\text{CVL}}-Acc_{\text{TDTS-TS}}$.

All methods show lower accuracy on CVL, consistent with the combined effect of residual recognition-domain shift (Section 5.4.1) and the greater irregularity of natural touching patterns. Notably, among the graph-theoretic segmentation algorithms, Left-Biased A* shows the largest drop, while Unbiased A* attains the highest absolute CVL accuracy, surpassing Dijkstra. This is consistent with Section 5.2.1: Left-Biased A*’s advantage on TDTS partly reflects that dataset’s left-leaning adhesion morphology (Chapter 3) rather than a general property of the method, and this advantage narrows once the directional prior no longer matches the data. Unbiased A*’s direction-agnostic design generalizes more consistently across both benchmarks.

### 5.5 Cross-paradigm comparison (U-Net)

To complete the cross-paradigm comparison, this section introduces a deep supervised segmentation baseline, U-Net, and examines its performance relative to the proposed graph-theoretic method under the same unified evaluation framework. Table 12 summarizes the end-to-end recognition accuracy of both paradigms on TDTS-TS and CVL, along with the cross-domain accuracy drop.

**Table 12:** Cross-paradigm comparison.

Category	Method	TDTS-TS	CVL	Cross-domain Drop
Reference ceiling	Single-character recognizer	98.03%	97.06%	—
Supervised segmentation	U-Net + same recognizer	95.90%	53.05%	−42.85
Unsupervised segmentation	Unbiased A* + same recognizer	92.68%	91.4%	−1.28

Note: The single-character recognizer is MRM on TDTS and the CVL recognizer on CVL, respectively; it quantifies the performance ceiling of the segmentation module. Cross-domain drop is meaningful only for the two-digit segmentation methods and is defined as CVL accuracy minus TDTS-TS accuracy.

On the synthetic TDTS data, U-Net exhibits the expected advantage of supervised learning. Relative to the single-character recognition ceiling (98.03%), U-Net’s end-to-end two-digit accuracy after segmentation is 95.90%, a segmentation loss of about 2.1 percentage points; Unbiased A* attains 92.68%, a segmentation loss of about 5.4 percentage points. This result is expected: U-Net has direct access to pixel-level ground truth during training and can fully fit TDTS’s synthetic adhesion pattern.

On the real-world CVL handwriting data, the two paradigms diverge fundamentally. The CVL single-character recognizer alone reaches 97.06%, yet after zero-shot transfer, U-Net’s two-digit accuracy collapses to 53.05%, a segmentation loss of 44 percentage points—directly indicating that the collapse originates from segmentation rather than recognition. By contrast, Unbiased A* maintains 91.4% on CVL, only 5.7 percentage points below the recognition ceiling. In terms of cross-domain drop, U-Net loses 42.85 percentage points versus Unbiased A*’s 1.28 percentage points—a difference exceeding one order of magnitude in cross-domain stability.

U-Net’s collapse on CVL is not an implementation error but a structural limitation of the supervised segmentation paradigm: U-Net requires large-scale pixel-level annotation for training, and unlike TDTS, real handwritten data cannot have its ground truth programmatically derived from generation parameters—it can only be manually labeled pixel by pixel, which is costly and subjective. While a programmatically generated dataset can provide labels at low cost, its synthetic distribution struggles to cover the diversity of real handwriting. Visualization results (Fig 11) further show that U-Net’s misclassifications concentrate near TDTS’s fixed fusion zone (x∈[13,19]): this positional prior becomes frozen into the convolutional weights during training, and at inference U-Net cannot recompute the corridor position from each image’s content the way the proposed cutting-domain method does (Section 5.4.2). Consequently, whenever a real adhesion point falls outside this fixed zone, cross-character pixel misclassification occurs there—strokes being misassigned to the opposite character—rather than merely a small shift in the cut boundary. Supervised segmentation thus faces a dual constraint of “annotation dependency” and “distribution coverage” in real-world document analysis, whereas the unsupervised graph-theoretic method proposed here adapts directly to new data through geometric rules and graph search, unconstrained by either.

**Fig 11:**
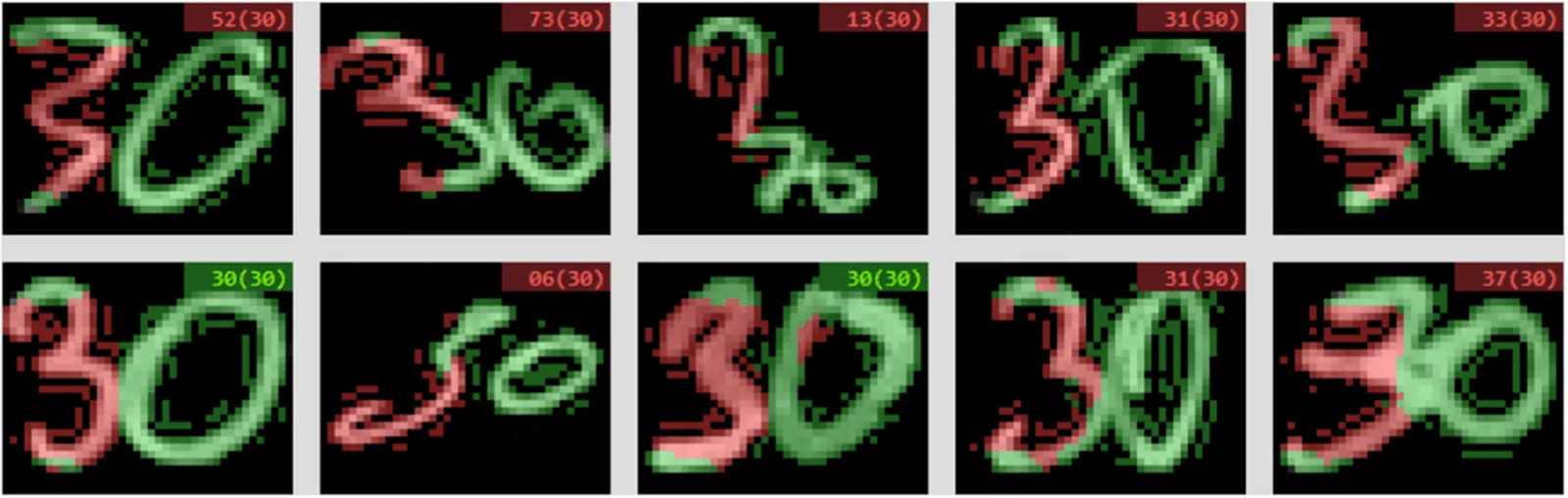
Visualization of U-Net’s segmentation predictions on CVL touching-digit pairs, showing cross-character pixel misclassification near the fixed fusion zone.

### 5.6 Chapter summary

This chapter systematically demonstrates the superiority of the cutting-domain-based graph-theoretic segmentation framework through comprehensive experiments. Key contributions are summarized as follows:

- **Establishment of a rigorous evaluation system:** By training the Mixed Recognition Model and implementing a unified pipeline, a fair and reliable benchmark is provided for touching digit segmentation research.
- **Verification of method superiority:** Experiments confirm that Dijkstra and A* algorithms significantly outperform traditional methods (VPM, WA) in accuracy. Left-Biased A* achieves the highest accuracy (93.13%), while Unbiased A* balances efficiency and accuracy optimally.
- **Revelation of key mechanism impacts:** Core findings include: connectivity constraints are essential for cutting domain effectiveness; 4-neighborhood growth yields slightly better accuracy due to stricter path constraints; and the denoising module is non-decisive under the current benchmark but valuable for extreme noise scenarios.
- **Provision of practical guidelines:** Algorithm and parameter recommendations are provided for diverse application scenarios—Dijkstra for high accuracy, A* variants for high efficiency, and 4-neighborhood growth for optimal segmentation precision.

This chapter not only empirically confirms the effectiveness of the proposed framework but also derives instructive methodological insights, laying a practical foundation for touching character segmentation and broader image segmentation research.

## 6 Conclusions and discussions

### 6.1 Research summary

This paper conducts a systematic review of the problem of handwritten digit segmentation from both data and methodological perspectives. Firstly, a standardized, category-balanced two-digit-touching-image dataset was constructed, providing a fair and controllable benchmark for algorithm evaluation. Secondly, the core innovative “cutting domain” framework was proposed, which decouples the image segmentation problem into two steps: constructing a structured low-cost search space (the cutting domain) via a threshold-free growth algorithm, and subsequently transforming the segmentation task into an optimal path search problem on a graph. Within this framework, the globally optimal Dijkstra’s algorithm and a family of direction-biased A* heuristic algorithms were implemented. Experimental results demonstrate that the proposed graph-theoretic segmentation method significantly outperforms traditional methods in overall two-character recognition accuracy. Among them, the Left-Biased A* algorithm achieved the highest accuracy of 93.1%. Ablation experiments verified the critical role of connectivity constraints and distilled design principles such as “algorithm-data characteristic matching,” proving the method’s robustness and generalization capability. Further validation on real-world CVL handwriting data confirmed this generalization capability (91.4% accuracy), while a cross-paradigm comparison revealed that a supervised U-Net baseline, despite matching performance on synthetic data, collapses under the same domain shift (53.05%).

### 6.2 Future work

Future work can be pursued in the following directions: (1) Exploring cutting domain generation methods that integrate semantic features to handle more complex adhesion patterns. (2) Extending the dataset to include more handwriting styles and multi-lingual characters, enhancing the benchmark’s universality. (3) Further optimizing algorithm efficiency by investigating lightweight and acceleration strategies to meet real-time application requirements. (4) Attempting to migrate the decoupled design principle of this framework to other image segmentation domains, such as medical image analysis or road extraction, to test its generalization potential.
